# The role of emotionality in the acquisition of new concrete and abstract words

**DOI:** 10.3389/fpsyg.2015.00976

**Published:** 2015-07-09

**Authors:** Pilar Ferré, David Ventura, Montserrat Comesaña, Isabel Fraga

**Affiliations:** ^1^Research Center for Behavior Assessment and Department of Psychology, Rovira i Virgili University, TarragonaSpain; ^2^Human Cognition Lab, CIPsi, School of Psychology, University of Minho, BragaPortugal; ^3^Cognitive Processes & Behavior Research Group, Department of Social Psychology, Basic Psychology and Methodology, University of Santiago de Compostela, Santiago de CompostelaSpain

**Keywords:** emotional words, concreteness, novel vocabulary learning, translation task, lexical decision

## Abstract

A processing advantage for emotional words relative to neutral words has been widely demonstrated in the monolingual domain (e.g., Kuperman et al., 2014). It is also well-known that, in bilingual speakers who have a certain degree of proficiency in their second language, the effects of the affective content of words on cognition are not restricted to the native language (e.g., Ferré et al., 2010). The aim of the present study was to test whether this facilitatory effect can also be obtained during the very early stages of word acquisition. In the context of a novel word learning paradigm, participants were trained on a set of Basque words by associating them to their Spanish translations. Words’ concreteness and affective valence were orthogonally manipulated. Immediately after the learning phase and 1 week later, participants were tested in a Basque go-no go lexical decision task as well as in a translation task in which they had to provide the Spanish translation of the Basque words. A similar pattern of results was found across tasks and sessions, revealing main effects of concreteness and emotional content as well as an interaction between both factors. Thus, the emotional content facilitated the acquisition of abstract, but not concrete words, in the new language, with a more reliable effect for negative words than for positive ones. The results are discussed in light of the embodied theoretical view of semantic representation proposed by Kousta et al. (2011).

## Introduction

During the last decade, studies devoted to the relationship between language and emotion have grown exponentially. A large body of research has focused on the effects of the emotional content of words on several cognitive processes, such as word recognition, attention, or memory (see Yiend, 2010; Citron, 2012; Talmi, 2013; Kuperman et al., 2014, for recent overviews). Although the results are not entirely consistent, there is now high consensus in that affectively valenced words show an advantage in processing with respect to neutral words, as revealed in word recognition tasks (e.g., lexical decision, Kousta et al., 2009; Kuperman et al., 2014), in naming tasks (Kuperman et al., 2014) or in memory tasks (e.g., Herbert et al., 2008; Talmi, 2013; Ferré et al., 2014), among others. Importantly, the effects of the affective content of words on cognition are not restricted to the native language. Indeed, they are also observed in the non-native languages of multilingual speakers who have a certain degree of proficiency in these languages (e.g., Ferré et al., 2010; Ponari et al., 2015; see also Caldwell-Harris, 2014, for a recent overview). A relevant question is whether the affective content of words has also an effect during the very early stages of foreign language acquisition. The present research addresses this issue by using a novel-word learning paradigm.

This paradigm has been typically used in the literature to identify the factors that facilitate the acquisition and consolidation of new words in the vocabulary. Usually these words belong to a foreign language, although they can also be pseudowords or very low frequency words in the native language (see De Groot, 2011, for an overview). Different training methods have been employed along with this paradigm. A popular method is paired-associate learning, in which the novel words are paired with their translations in the first language (L1, e.g., De Groot and Keijzer, 2000; Comesaña et al., 2009, 2012; Altarriba and Basnight-Brown, 2011a; Geukes et al., 2015). Other methods consist of associating new words and their concepts by means of pictures (Yu and Smith, 2007; Comesaña et al., 2009, 2012; Palmer and Havelka, 2010), or presenting novel words together with their definitions (e.g., Clay et al., 2007; Palmer et al., 2013; Tamminen and Gaskell, 2013).

To test the effectiveness of the above methods, researchers have relied on different tasks, depending on the particular goal of the study. For instance, researchers interested on assessing the number of words acquired in a prior training episode have mostly applied cued recall tasks (i.e., to produce a word in response to a specific cue, De Groot, 2011). In particular, a widely used procedure has been to ask participants to produce either the L1 translation equivalents of the novel words acquired in the foreign language (the so-called backward translation, De Groot and Keijzer, 2000; Kaushanskaya and Marian, 2009; Farley et al., 2012), or the translation equivalents in the foreign language in response to L1 words (i.e., forward translation, De Groot and Keijzer, 2000). Other studies have tried to determine the state of the new vocabulary, assessing whether the recently learned words behave as familiar words in different tasks, such as the lexical decision task (LDT; Elgort, 2010; Palmer et al., 2013), and exploring what different types of knowledge the learner has acquired about the new words.

The main focus of interest in this line of research concerns semantic knowledge. Thus, researchers have investigated whether effective links between the novel words and the corresponding concepts are established. To do so, they have used different approaches: some authors have tested whether the performance in different tasks is affected by the semantic characteristics of the new words, such as their concreteness (De Groot and Keijzer, 2000; Kaushanskaya and Rechtzigel, 2012; Palmer et al., 2013). Others have investigated whether specific semantic effects can be obtained with these new words. Among them, there are the semantic priming effect (Elgort, 2010; Tamminen and Gaskell, 2013), the Stroop effect (Geukes et al., 2015), and the interference effect produced by words semantically related to the correct translation in a translation recognition task (Comesaña et al., 2009, 2012; Altarriba and Basnight-Brown, 2011a; Poarch et al., 2014).

The results of the above research have revealed that different variables can modulate the outcome of novel-word learning procedures. A relevant factor is the training method used. Thus, although direct access from the new words to concepts seems to be achieved with methods based on lexical associations (see Comesaña et al., 2012; Poarch et al., 2014, for recent reviews), stronger lexico-semantic links are found with methods that emphasize conceptual mediation, such as those based on picture-L2 associations (Comesaña et al., 2009; Dobel et al., 2010), or on the presentation of definitions together with the novel words (Tamminen and Gaskell, 2013). Another variable to consider is the time elapsed between the acquisition phase and the testing phase. Most studies assess performance in two different moments: shortly after learning and then, again, after a period time (i.e., several hours or days). These studies reveal that there is a decrease in performance between the two sessions, suggesting that some information has been forgotten (e.g., De Groot and Keijzer, 2000) and that a consolidation period seems to be required for several effects to appear (e.g., lexical competition with similar previously known words, Davis et al., 2008, or semantic interference, Comesaña et al., 2009).

Of relevance for the present study is the type of word, another variable that has shown to affect new vocabulary training results. Researchers have focused on cognate status and concreteness, demonstrating that cognate words are learned faster than non-cognate words (De Groot and Keijzer, 2000; Tonzar et al., 2009; Comesaña et al., 2012), and that concrete words are learned faster than abstract words (De Groot and Keijzer, 2000; Altarriba and Basnight-Brown, 2011a; Kaushanskaya and Rechtzigel, 2012; Palmer et al., 2013). In contrast, the effect of the affective properties of words, in spite of their widespread study in other fields, has been scarcely addressed in this literature.

To our knowledge, the only study that has dealt with the effect of the emotional content of novel words on their acquisition is that of Altarriba and Basnight-Brown (2011a). In this study, native speakers of English learned the Spanish translations of concrete, abstract and emotion words (i.e., words that label an emotion, -scared), by associating them to their English translation equivalents. After training, participants were tested in a Stroop task and in a backward translation recognition task. In the Stroop task, participants were required to press a key denoting the ink color of the Spanish words presented. The authors found faster responses to emotion words than to concrete and abstract words. This result contrasts with the typical emotional Stroop effect repeatedly found in both people’s native language and in bilinguals’ second language [i.e., faster response times (RTs) for neutral words than for affectively charged words, which are considered a result of the attentional capture by the latter, e.g., Eilola et al., 2007]. Concerning the backward translation recognition task, participants were presented with pairs including a new learned Spanish word and an English word that could be either its translation equivalent, an English word semantically related to the correct translation, or an unrelated word. With respect to incorrect translations, concrete, abstract and emotion words revealed the same pattern of results. That is, participants took longer times to reject as incorrect translations semantically related words than unrelated words (i.e., a semantic interference effect), suggesting that the link between the novel words and concepts had been established during training. Importantly, regarding correct translations, participants responded slower to emotion words than to abstract or concrete ones, the later words showing the shortest RTs. The finding of slower RTs for emotion words than for neutral words is also at odds with all the literature reporting an advantage for affectively valenced words in tasks such as lexical decision (e.g., Kousta et al., 2009), naming (e.g., Kuperman et al., 2014), or free recall (e.g., Altarriba and Bauer, 2004; Herbert et al., 2008; Ferré et al., 2014). In light of these findings, Altarriba and Basnight-Brown (2011a) concluded that recently acquired emotion words do not have the same properties as familiar words. They argued that the semantic representation of the former is less rich than that of the latter. The reason would be that only familiar words would have been experienced in emotional contexts over a long period of time.

As stated above, the study of Altarriba and Basnight-Brown (2011a) is the only one that has addressed the effects of emotional content on novel-word learning, making an interesting contribution to the field. However, the authors only tested negatively valenced emotion words. As past research in word processing and memory suggests that the experimental findings obtained with negative words do not always converge with those obtained with positive words (e.g., Estes and Adelman, 2008; Herbert et al., 2008), it is relevant to compare these two types of valenced words in vocabulary acquisition paradigms. On the other hand, it should be mentioned that Altarriba and Basnight-Brown (2011a) did not completely disentangle the effects of emotional content and concreteness in their results, since some of the words included in the abstract condition might have been affectively charged too (e.g., virtue). In order to elucidate which effects are produced by emotional content and which are produced by concreteness, an orthogonal manipulation of both variables should be done.

The orthogonal manipulation of emotional content and concreteness is, in fact, relevant in light of a recent theoretical proposal of Kousta et al. (2011). These authors have proposed an embodied theoretical view of semantic representation according to which sensory-motor information would be central to the representation and processing of concrete words, whereas affective information would be more relevant in the representation of abstract words. Thus, abstract words would be more affectively loaded than concrete words. Importantly, Kousta et al. (2011) posited that the emotional content would play an important role during language acquisition, facilitating the acquisition of abstract lexical concepts and their labels during childhood. To confirm that, these researchers conducted a regression analysis on a large set of words and observed that, for abstract words, valence and age of acquisition were related by a U-shaped function. That is, abstract emotional words seem to be acquired before abstract neutral words.

If emotional content can facilitate the acquisition of abstract words, it might be possible that this modulation is not only observed when children acquire their first language, but also when adult people learn vocabulary in a new language. This is the prediction we aimed to test in the present work by orthogonally manipulating emotional content and concreteness. We used a novel word learning paradigm in which participants learned the Basque translations of concrete and abstract Spanish words that were positive, negative or neutral, by associating them to their Spanish translations. (Basque is an ancient pre-Indo-European language spoken in a small area in the eastern part of the Pyrenees.) In particular, we investigated whether the acquisition of abstract words in a new language is modulated by their emotional content to a greater extent than the acquisition of concrete words.

To sum up, the aim of the present work was to shed further light on the characteristics of the words that can facilitate their acquisition in a paired-associate word learning paradigm. We used the paired-associate learning task because it has been commonly employed in foreign-language training programs (De Groot and Keijzer, 2000; De Groot, 2011). Furthermore, this procedure allows the inclusion of both concrete and abstract words as experimental materials, in contrast to other paradigms such as the association of novel words to pictures, which can be used only with concrete words.

Therefore, participants learned a set of novel Basque words, by associating them to their Spanish translations. Then, they were tested in two different tasks: a go/no-go LDT and a backward translation task, both immediately after acquisition and 1 week later. These two testing sessions were included to assess long-term word retention. We used the LDT to explore whether emotional content produces an advantage in the recognition of recently trained foreign words, as has been observed with words in the native language (Kousta et al., 2009; Kuperman et al., 2014) as well as with words in the L2 of proficient bilinguals (Ponari et al., 2015). Regarding the translation task, it was used as a measure of the participants’ success in linking the novel Basque words to their referents in L1. In fact, the translation task is the most widely employed in the literature to assess the success of training procedures in terms of the number of learned words (De Groot and Keijzer, 2000; De Groot, 2011; Kaushanskaya and Rechtzigel, 2012).

Taking into consideration the results of past studies in novel vocabulary acquisition (e.g., De Groot and Keijzer, 2000), we expected both a concreteness advantage and a session effect (i.e., better performance during the first session than during the second session). Importantly, if emotional content modulates the acquisition of new vocabulary, we would expect better performance with emotional words than with neutral words. Furthermore, if the emotional content mainly facilitates the acquisition of abstract words, as could be predicted from the proposal of Kousta et al. (2011), we should expect an interaction between emotional content and concreteness. Finally, taking into account that, in order to decide whether a given string of letters is a Basque word (i.e., the LDT task), participants can rely on the familiarity with its form, whereas in order to produce the Spanish equivalent, they have to rely on the links established during acquisition between the translation equivalents, we expected a better performance in the LDT than in the translation task.

## Materials and Methods

### Participants

Fifty undergraduate students of Psychology (41 women) from the University Rovira i Virgili (Tarragona, Spain) took part in the experiment (*M*_age_ = 21.8, SD = 4.4). All of them were highly proficient and balanced Catalan-Spanish bilinguals and had not any knowledge of the Basque language. They had normal or corrected-to-normal vision and all of them received a course credit for their participation.

### Materials

The stimuli used in the present study comprised a set of 48 Spanish words and their Basque translations. The 93.4% of the words were nouns and the 6.4% were adjectives. Spanish words were obtained from two normative databases: the Spanish Adaptation of ANEW (Redondo et al., 2007), and the Affective Norms of Ferré et al. (2012). The Basque translations were obtained from an on-line Spanish-Basque dictionary published by the Autonomous Basque Government (Elhuyar Online Dictionary of Spanish and Basque, 2003) and were checked by a proficient bilingual of Basque and Spanish. Words were divided into six sets: concrete positive words, concrete negative words, concrete neutral words, abstract positive words, abstract negative words, and abstract neutral words (see Data Sheet 1).

The Spanish words in the critical conditions were matched in several variables that can affect word processing (see **Table 1**). The values of frequency of use, length, number of lexical neighbors, mean bigram frequency, concreteness, and imageability were obtained from B-Pal (Davis and Perea, 2005). We also considered the degree of orthographic similarity (OS) between the Spanish words and their Basque translations, by using the NIM database (Guasch et al., 2013). NIM computes the index of van Orden (1987), which ranges from 0 (not similar at all) to 1 (exactly the same). The words selected had values of OS lower than 0.5 (i.e., they were non-cognates) to avoid influences of cross-language similarities on novel word acquisition and processing. As participants were bilinguals of Catalan and Spanish, and they had some knowledge of English, the OS between the novel Basque words and their translations in Catalan and English were also considered. Additionally, the OS between Catalan and Spanish was taken into account to guarantee an equal distribution across conditions of cognates between these two languages. Finally, in order to discard differences in the difficulty of the Basque words across the different experimental conditions, we obtained the Spanish frequency of the bigrams in the Basque words from B-Pal.

**Table 1:** Mean of the lexical characteristics of the experimental stimuli (standard deviation in parentheses).

	Concrete positive	Concrete negative	Concrete neutral	Abstract positive	Abstract negative	Abstract neutral
Conc	5.8 (0.5)	5.9 (0.6)	5.8 (0.6)	3.5 (0.3)	3.4 (0.4)	3.3 (0.5)
Imag	5.9 (0.4)	5.8 (0.6)	6.4 (0.4)	3.4 (0.5)	3.1 (0.5)	3.2 (0.7)
Val	7.3 (0.8)	2.1 (0.5)	4.7 (0.2)	7.8 (0.4)	1.9 (0.2)	4.6 (0.2)
Aro	6.3 (0.6)	6.8 (0.4)	3.8 (0.5)	6.7 (0.5)	6.7 (0.4)	4.3 (0.5)
Spanlength	7.0 (1.5)	6.5 (1.9)	7.5 (1.2)	8.4 (2.1)	7.2 (2.3)	8.4 (1.7)
LogFreq	1.3 (0.6)	1.2 (0.6)	0.7 (0.4)	1.4 (0.6)	1.3 (0.4)	1.3 (0.2)
Neighbors	0.5 (0.5)	0.7 (1.0)	0.5 (1.1)	0.2 (0.5)	0.7 (1.0)	0.4 (0.5)
Spanbigfreq	2.3 (0.4)	2.3 (0.4)	2.5 (0.2)	2.6 (0.2)	2.4 (0.4)	2.4 (0.4)
Basqlength	8.2 (1.8)	6.9 (1.4)	7.5 (1.4)	8.8 (0.9)	8.0 (2.9)	7.8 (1.2)
Basqbigfreq	1.4 (0.4)	1.2 (0.4)	1.8 (0.5)	1.3 (0.4)	1.4 (0.6)	1.4 (0.3)
OS SpaBas	0.1 (0.1)	0.1 (0.1)	0.1 (0.0)	0.1 (0.1)	0.1 (0.1)	0.1 (0.1)
OS CatBas	0.1 (0.1)	0.2 (0.1)	0.1 (0.0)	0.1 (0.1)	0.1 (0.1)	0.1 (0.1)
OS EngBas	0.1 (0.1)	0.1 (0.1)	0.1 (0.0)	0.1 (0.1)	0.1 (0.1)	0.1 (0.0)
OS SpaCat	0.6 (0.3)	0.7 (0.2)	0.5 (0.3)	0.6 (0.3)	0.6 (0.2)	0.8 (0.1)

*Concr, concreteness (from 1 to 7); Imag, imageability (from 1 to 7); Val, valence (from 1 to 7); Aro, arousal (from 1 to 7); Spanlength, mean number of letters for the Spanish words; LogFreq, mean log frequency per million words; Neighbors, mean number of substitution neighbors of the Spanish words; Spanbigfreq, mean bigram frequency of the Spanish words; Basqlength, mean number of letters of the Basque words; Basqbigfreq, mean bigram frequency of Spanish in the Basque words; OS SpaBas, orthographic similarity between the Basque words and their Spanish translations; OS CatBas, orthographic similarity between the Basque words and their Catalan translations; OS EngBas, orthographic similarity between the Basque words and their English translations and OS SpaCat, orthographic similarity between the Spanish words and their Catalan translations.*

We conducted a 2 (concreteness) × 3 (emotional content) ANOVA on the above mentioned variables. The analysis revealed, as expected, a significant effect of concreteness on words’ concreteness, *F*(1,42) = 290.80, MSE = 0.24, *p* < 0.001, η^2^ = 0.87 and imageability, *F*(1,42) = 333.95, MSE = 0.28, *p* < 0.001, η^2^ = 0.89. That is, concrete words had higher values of concreteness (*M* = 5.83) and imageability (*M* = 6.0) than abstract words (*M* = 3.40 and *M* = 3.23 for concreteness and imageability, respectively). There was also a significant effect of emotional content on both valence, *F*(2,42) = 613.03, MSE = 0.20, *p* < 0.001, η^2^ = 0.97 and arousal, *F*(1,42) = 160.77, MSE = 0.22, *p* < 0.001, η^2^ = 0.88. Pairwise Bonferroni comparisons showed that positive (*M* = 7.54), negative (*M* = 1.98), and neutral words (*M* = 4.67) were significantly different concerning valence (*p* < 0.001). In addition, positive and negative words were matched in extremity (i.e., the difference between the valence of emotional words and the average valence value of neutral words), *t*(30) = 0.93, *p* = 0.36. With respect to arousal, both positive (*M* = 6.50) and negative words (*M* = 6.74) were more arousing than neutral words (*M* = 4.04, *p* < 0.001), although there was not any difference between positive and negative words in that variable (*p* = 0.16). Importantly, the ANOVA revealed that there was not any effect of either concreteness or emotional content on frequency, length, number of lexical neighbors, or mean bigram frequency of the Spanish words as well as of the Basque words in Spanish. Similarly, neither concreteness nor emotional content had any effect on the different measures of OS computed (all *F*s < 1.99).

We also constructed a set of 48 Basque pseudowords to be used in the LDT. These pseudowords were obtained from the Wuggy software (Keuleers and Brysbaert, 2010) and were matched to the Basque experimental words in length, mean bigram frequency, and number of lexical neighbors in Basque (data taken from E-Hitz, Perea et al., 2006).

### Procedure

The experimental procedure followed the ethical guidelines of the Faculty of Sciences of Education and Psychology of the University Rovira i Virgili. In addition, participants signed an informed consent before starting the experiment. Participants were individually tested in separate soundproof booths. The experiment consisted of two sessions. The first session began with a learning phase. During this phase, participants were presented with pairs of Basque words and their Spanish translations in six blocks of eight words. Five different random presentation orders were created, to which each participant was randomly assigned. Each block of eight pairs was presented three times using Microsoft-Powerpoint. The first time, each block was displayed visually during 2 min while participants also heard the pairs of Basque-Spanish translations. They were asked to study these pairs. During the second presentation, the same six blocks appeared. The presentation of each block was as follows: initially, only the first word of the eight pairs (i.e., the Basque word) was displayed, and participants were asked to try to think of their Spanish translation. After 45 s, the Spanish translations appeared together with the Basque words. They remained on the screen for 2 min and participants were asked to study again the pairs. Then, the following block was displayed. During the third presentation, the same six blocks (including the eight Basque-Spanish translation pairs) were presented during 1 min and participants were asked again to study them.

Immediately after the learning phase, participants performed a go-no go LDT. Participants were presented with the 48 Basque words mixed with 48 Basque pseudowords. They had to decide whether each sequence corresponded to a previously learned Basque word or not, by pressing the “yes” button of a keypad with the preferred hand. If they did not recognize the string as a Basque word, they had to refrain from responding. We chose this approach in order to make the task less demanding, since an advantage of the go/no-go procedure with respect to the standard LDT (i.e., faster and more accurate responses) has been reported in the literature (see Gómez et al., 2007). Presentation of the stimuli and recording of RTs and errors were controlled by using the DMDX software (Forster and Forster, 2003). On each trial, the sequence was as follows: first, a fixation point (i.e., “+”) appeared in the middle of the screen for 500 ms. Immediately afterward, the letter string was presented until participants responded or for a maximum of 2000 ms. The inter-trial interval (ISI) was 1000 ms.

When the LDT was finished, participants performed a translation task. They were presented with a sheet of paper containing the 48 Basque words (in a different order with respect to the acquisition) and they were given 10 min to try to produce as many Spanish translations as they could (i.e., backward translation). They were encouraged to guess. We used this direction of translation because forward direction (i.e., to produce the learned Basque words in response to the Spanish words) has been demonstrated to be more difficult for people that are at the initial stages of learning a foreign language (De Groot and Keijzer, 2000; De Groot, 2011). After finishing the translation task, participants were requested to come back to the laboratory the following week in order to continue with the experiment. They were not informed of the content of the second experimental session.

The second session was conducted 1 week after the first one. Participants came back to the laboratory and were administered with the same LDT and the same translation task in the same order as in the first session. When the experiment was finished, participants were thanked for their participation and debriefed.

## Results

### Lexical Decision Task

Incorrect responses and RTs lower than 200 ms were excluded from the latency analysis. There was no upper limit for RTs. Values falling more than 2 SD from the mean for a given participant in all conditions were also removed. As a result, 0.5% of the data was removed in the first session. In the second session, the percentage of rejected data was 0.3%.

The results are shown in **Table 2**. The analyses were restricted to the responses to Basque words (i.e., pseudowords were not considered in the analyses). We conducted separate ANOVAs by participants and by items on RT and on Accuracy (i.e., the percentage of Basque words correctly identified). The analyses included the factors Session (Session 1 vs. Session 2), Concreteness (concrete vs. abstract words) and Emotional content (positive, negative, and neutral words). All were within-participant factors in the analysis by participants. In the analysis by items, Session was a within-items factor and both Concreteness and Emotional content were between-items factors.

**Table 2:** Results of the Lexical decision task -LDT- (mean of RTs and mean of the percentage of correctly identified Basque words-Accuracy) and the Translation task (mean of the percentage of correctly translated Basque words-Accuracy).

	Session 1			Session 2		
Experimental condition	LDT -RTs	LDT -accuracy	Translation -accuracy	LDT -RTs	LDT -accuracy	Translation -accuracy
Concr positive words	1,018.9 (169.7)	79.0 (17.5)	63.7(24.1)	963.3 (184.4)	75.2 (16.5)	39.2 (20.9)
Concr negative words	981.6 (183.3)	73.6 (20.9)	58.7 (25.5)	973.3 (181.8)	72.2 (18.7)	35.5 (21.4)
Conc neutral words	958.4 (171.4)	76.8 (16.6)	60.2 (25.8)	968.4 (155.7)	77.0 (15.4)	36.2 (23.9)
Abst positive words	1,107.9 (248.8)	71.0 (20.4)	27.2 (22.1)	1,078.5 (172.9)	63.2 (17.9)	12.7 (15.3)
Abst negative words	948.1 (163.3)	80.7 (17.1)	46.0 (27.9)	956.6 (1729.9)	75.5 (19.6)	19.0 (18.1)
Abst neutral words	1,073.2 (225.3)	66.4 (20.3)	21.7 (23.8)	1,026.1 (180.9)	62.0 (18.4)	7.5 (9.8)

*Standard deviation is presented in parentheses.*

*Concr, concrete; Abst, abstract.*

The ANOVA on RT only included correct responses. This analysis revealed a main effect of concreteness, significant only in the analysis by participants, *F*_1_(1,49) = 26.26, MSE = 16911.84, *p* < 0.001, η^2^ = 0.35, *F*_2_(1,42) = 2.64, MSE = 27971.19, *p* = 0.11, η^2^ = 0.06, revealing that participants responded faster to concrete words (*M* = 977.33) than to abstract words (*M* = 1031.74). Emotional content also reached statistical significance in the by-participants analysis, *F*_1_(2,98) = 15.78, MSE = 18957.45, *p* < 0.001, η^2^ = 0.24, *F*_2_(2,42) = 1.81, MSE = 27971.19, *p* = 0.18, η^2^ = 0.08. Bonferroni *post hoc* tests showed that participants took less time to identify negative words (*M* = 964.90) than both positive words (*M* = 1042.18, *p* < 0.001) and neutral words (*M* = 1006.52, *p* < 0.05). Furthermore, the difference between positive and neutral words approached significance (*p* = 0.07). Importantly, the interaction between concreteness and emotional content was also significant in the analysis by participants, *F*_1_(2,98) = 9.96, MSE = 24097.99, *p* < 0.001, η^2^ = 0.17, *F*_2_(2,42) = 1.26, MSE = 27971.19, *p* = 0.29, η^2^ = 0.06. This interaction revealed that the effect of the emotional content was restricted to abstract words, where negative words were responded faster (*M* = 952.36) than both positive (*M* = 1093.22, *p* < 0.001) and neutral words (*M* = 1049.64, *p* < 0.01), whereas there was not any difference between positive and neutral words (*p* = 0.37). Neither the effect of session nor the remaining interactions reached statistical significance (all *F*s < 1.96).

The ANOVA on Accuracy showed a significant effect of session, *F*_1_(1,49) = 6.56, MSE = 322.02, *p* < 0.05, η^2^ = 0.12, *F*_2_(1,42) = 19.71, MSE = 24.54, *p* < 0.001, η^2^ = 0.32, indicating that participants were more accurate in the first session (*M* = 74.60) than in the second session (*M* = 70.84). Concreteness was also significant in the analysis by participants, *F*_1_(1,49) = 18.12, MSE = 281.78, *p* < 0.001, η^2^ = 0.27, *F*_2_(1,42) = 2.23, MSE = 362.38, *p* = 0.14, η^2^ = 0.56, as participants showed a higher accuracy with concrete words (*M* = 75.64) than with abstract words (*M* = 69.80). Emotional content reached significance too in the analysis by participants, *F*_1_(2,98) = 4.56, MSE = 280.96, *p* < 0.05, η^2^ = 0.08, *F*_2_(2,42) = 0.66, MSE = 362.38, *p* = 0.52, η^2^ = 0.03. *Post hoc* comparisons revealed that participants were more accurate with negative words (*M* = 75.50) than with neutral words (*M* = 70.55, *p* < 0.05), whereas there was not any difference in accuracy between positive (*M* = 72.11) and neutral words (*p* = 0.13). Finally, as in RT, the interaction between concreteness and emotional content was significant in the analysis by participants, *F*_1_(2,98) = 16.12, *p* < 0.001, MSE = 288.07, η^2^ = 0.25, *F*_2_(2,42) = 2.11, MSE = 362.38, *p* = 0.13, η^2^ = 0.09. This interaction indicated that the effect of the emotional content was again restricted to abstract words: participants were more accurate in responding to negative abstract words (*M* = 78.01) than to their positive (*M* = 67.12, *p* < 0.001) and neutral counterparts (*M* = 64.19, *p* < 0.001). There was not any difference between positive and neutral abstract words (*p* = 0.51). No other interaction reached statistical significance (all *F*s < 2.26).

### Translation Task

Response time data were not obtained in this task. The percentage of Basque words correctly translated to Spanish were collected (Accuracy, see **Table 2**). We conducted separate ANOVAs by participants and by items on that measure including the same factors as in the analyses of lexical decision data. The analyses revealed a main effect of session, *F*_1_(1,49) = 133.17, MSE = 512.63, *p* < 0.001, η^2^ = 0.73, *F*_2_(1,42) = 397.11, MSE = 7.20, *p* < 0.001, η^2^ = 0.90, as participants produced a higher percentage of correct translations in the first session (*M* = 46.29) than in the second session (*M* = 24.96). Furthermore, participants performed better with concrete words (*M* = 48.87) than with abstract words (*M* = 22.38), *F*_1_(1,49) = 309.19, MSE = 340.69, *p* < 0.001, η^2^ = 0.86, *F*_2_(1,42) = 29.27, MSE = 147.62, *p* < 0.001, η^2^ = 0.41. The emotional content reached statistical significance in the analysis by participants, *F*_1_(2,98) = 11.38, MSE = 299.34, *p* < 0.001, η^2^ = 0.19, *F*_2_(2,42) = 0.92, MSE = 147.62, *p* = 0.41, η^2^ = 0.04. Pairwise comparisons revealed that both positive (*M* = 35.75, *p* < 0.05) and negative words (*M* = 39.69, *p* < 0.001) were more accurately translated than neutral words (*M* = 31.44). The difference between positive and negative words approached significance (*p* = 0.07). The three simple interactions were significant too. The interaction between session and concreteness, *F*_1_(1,49) = 5.96, MSE = 190.49, *p* < 0.05, η^2^ = 0.11, *F*_2_(1,42) = 6.69, MSE = 7.20, *p* < 0.05, η^2^ = 0.14, showed that the size of the advantage for concrete words over abstract words was higher in the first session (*M* = 29.25) than in the second session (*M* = 23.75). Besides, it revealed that the decrease in performance between the first session and the second session was higher for concrete words (Mean decrease = 24.08) than for abstract words [Mean decrease = 18.58, *t*(49) = 2.44, *p* < 0.05]. The interaction between session and emotional content also reached statistical significance, *F*_1_(2,98) = 7.07, MSE = 86.85, *p* < 0.005, η^2^ = 0.13, *F*_2_(2,42) = 3.41, MSE = 7.20, *p* < 0.05, η^2^ = 0.14. *Post hoc* comparisons showed that, although negative words (Mean performance in Session 1 = 52.37, Mean performance in Session 2 = 27.00) were better translated than neutral words (Mean performance in Session 1 = 41.00, Mean performance in Session 2 = 21.87) in both sessions (*p* < 0.001 and *p* < 0.05 for the first and second session, respectively), their advantage over positive words (Mean performance in Session 1 = 45.50, Mean performance in Session 2 = 26.00) was restricted to the first session (*p* < 0.01). Concerning positive words, although they failed to show a significant difference with respect to neutral words, they tended to show an advantage over them in both the first (*p* = 0.07) and the second session (*p* = 0.08). In addition, the interaction between concreteness and emotional content, significant in the analysis by participants, *F*_1_(2,98) = 23.49, MSE = 237.10, *p* < 0.001, η^2^ = 0.32, *F*_2_(2,42) = 1.58, MSE = 147.62, *p* = 0.22, η^2^ = 0.07, showed that the enhancing effect of the emotional content was restricted to abstract words. Thus, negative abstract words (*M* = 32.50) were better translated than both their positive (*M* = 20.00, *p* < 0.001) and neutral counterparts (*M* = 14.62, *p* < 0.001). Positive abstract words were also better translated than neutral abstract words (*p* < 0.05). Finally, the triple interaction between session, concreteness and emotional content was also significant, *F*_1_(2,98) = 8.37, MSE = 85.79, *p* < 0.001, η^2^ = 0.15, *F*_2_(2,42) = 3.69, MSE = 7.20, *p* < 0.05, η^2^ = 0.15. *Post hoc* analyses revealed that, during the first session, the facilitatory effect of emotional content was observed only in abstract words. In particular, negative abstract words were better translated than both their neutral (*p* < 0.001) and positive counterparts (*p* < 0.001). Furthermore, although there was a trend for positive abstract words to be better translated than neutral ones, this difference did not reach statistical significance (*p* = 0.09). Concerning the second session, the pattern was very similar, with an effect of the emotional content restricted to abstract words. Pairwise comparisons revealed that negative abstract words were better translated than both positive (*p* < 0.05) and neutral words (*p* < 0.001). Likewise, positive abstract words were better translated than neutral ones (*p* < 0.05).

To further explore the differences between sessions, we computed the magnitude of the emotional effect in abstract words (i.e., we subtracted the percentage of neutral words correctly translated to the percentage of emotional words correctly translated). Concerning positive abstract words, the magnitude of the effect was very similar in both sessions (*M* = 5.50 and *M* = 5.25 for the first and second session, respectively). Conversely, negative abstract words showed a significant decrease in the magnitude of the emotional effect between the first (*M* = 24.25) and the second session (*M* = 12.50), *t*_1_(49) = 4.88, *p* < 0.001, *t*_2_(7) = 1.96, *p* = 0.09. Similarly, the difference between positive and negative abstract words correctly translated was larger in the first session (*M* = 18.75) than in the second one (*M* = 6.25), *t*_1_(49) = 4.81, *p* < 0.001, *t*_2_(7) = 4.28, *p* < 0.005.

Finally, in order to ascertain whether participants’ accuracy was affected by the type of task, we compared the percentage of correct responses in both tasks. We conducted an ANOVA including “Task” and “Session” as factors. This analysis revealed a main effect of “Task,” *F*_1_(1,49) = 157.31, MSE = 437.41, *p* < 0.001, η^2^ = 0.76, *F*_2_(1,47) = 594.53, MSE = 234.64, *p* < 0.001, η^2^ = 0.93 as well as of “Session,” *F*_1_(1,49) = 108.48, MSE = 72.52, *p* < 0.001, η^2^ = 0.69, *F*_2_(1,47) = 200.43, MSE = 14.21, *p* < 0.001, η^2^ = 0.81. These results showed that accuracy was higher in the LDT (*M* = 72.72) than in the Translation task (*M* = 35.62). Furthermore, there was a better performance during the first session (*M* = 60.44) than during the second session (*M* = 47.90). The interaction between both factors also reached statistical significance, *F*_1_(1,49) = 58.06, MSE = 66.59, *p* < 0.001, η^2^ = 0.54, *F*_2_(1,47) = 24.79, MSE = 19.99, *p* < 0.001, η^2^ = 0.34. *Post hoc* comparisons revealed that the decrease in accuracy when comparing the first session and the second session was larger for the translation task (*M* = 21.33) than for the LDT task (*M* = 3.75), *t*_1_(49) = 7.61, *p* < 0.001, *t*_2_(47) = 4.98, *p* < 0.001.

## Discussion

The aim of the present study was to test whether the affective content of words has a facilitatory effect during the early stages of a foreign language acquisition. We also investigated whether this effect was modulated by words’ concreteness. To do that, participants were trained on a set of Basque words by associating them to their Spanish translations. Immediately after the learning phase and 1 week later, they were tested in a go-no go LDT and in a backward translation task. Although performance, in terms of accuracy, was higher in the LDT than in the translation task, the pattern of results was very similar across tasks as well as over time: apart from a main effect of concreteness and emotional content, there was an interaction between both factors, indicating that the emotional content facilitated the acquisition of abstract rather than concrete words in a new language. Overall, the effect was more reliable for negative words than for positive words. Finally, even though there was a significant loss of information over time, which was higher for the translation task than for the LDT, the pattern of findings was very similar in both sessions.

This work adds to the literature in novel-word training showing that some word types are more easily acquired than others. Thus, apart from cognate status (De Groot and Keijzer, 2000; Tonzar et al., 2009; Comesaña et al., 2012) and concreteness (De Groot and Keijzer, 2000; Altarriba and Basnight-Brown, 2011a; Kaushanskaya and Rechtzigel, 2012; Palmer et al., 2013, and the present study), it is apparent that emotional content facilitates word acquisition during the early stages of foreign language learning. In this field of research, this is the first time that a facilitatory effect of emotional content is reported. Importantly, the effect is restricted to abstract items, suggesting that the words that are harder to learn are those obtaining more benefits from their affective content. Although these results were only significant in the participant analyses, we would like to note that they are consistent with the proposal of Kousta et al. (2011), according to which the emotional content would play a more relevant role in the representation and processing of abstract words than in the representation and processing of concrete words.

Further studies should be conducted to establish the extent to which this pattern of findings can be generalized to other sets of items, especially because this facilitatory effect for emotional words was not observed in the study of Altarriba and Basnight-Brown (2011a). In that work, the authors failed to obtain either the emotional Stroop effect or an advantage in the translation recognition task for recently acquired Spanish emotion words. Although the reasons for this discrepancy are unclear, it is worth mentioning that the different methodological approaches limit the comparison between those two studies. On the one hand, unlike the present study, Altarriba and Basnight-Brown (2011a) did not orthogonally manipulate concreteness and emotional content. On the other, these authors compared concrete and abstract words to emotion words (i.e., words labeling an emotion, -scared), whereas we mostly used emotion-laden words (i.e., words that do not refer directly to emotions but have emotional connotations, for example *success*). Some researchers have suggested that these two word types can be processed differently (e.g., Pavlenko, 2008), although the few studies which have addressed this distinction have only found slight differences between them (e.g., Altarriba and Basnight-Brown, 2011b). Thus, further research is needed in which the contribution of concreteness and emotional content to the initial stages of word acquisition is evaluated separately for emotion and emotion-laden words.

As stated above, our findings support the proposal of Kousta et al. (2011). These authors argued that whereas sensory-motor information would be central to the representation and processing of concrete words, affective information would be more relevant in the representation of abstract words. In fact, it would be through emotionality that abstract words would become embodied, as one of the functions of emotion is to initiate approach and avoidance behavior (e.g., Lang, 1995). Thus, one might expect strong sensory-motor effects for abstract words as well, if effects are mediated by emotion, as indicated for instance by the startle reflex (see Herbert and Kissler, 2010; Herbert et al., 2011 for evidence of modulation of the startle reflex by the emotional content of words)^1^. Importantly, as Sheikh and Titone (2013) have recently pointed out, a prediction from the embodied approach to semantic representation of Kousta et al. (2011) is that the contribution of emotionality to word processing would be more likely in conditions in which there are no other sources of information (i.e., sensorimotor) that can facilitate processing. This is the case for abstract words.

During the last years, several lexical decision studies have addressed the possible modulation of emotional effects by concreteness in the native language of adult speakers during word processing. This research has yielded mixed findings. Thus, whereas Kanske and Kotz (2007) failed to find any interaction between emotional content and concreteness, Palazova et al. (2013) did obtain it, although in this case emotional abstract words showed a disadvantage, rather than an advantage, with respect to their neutral counterparts. These inconsistencies might be explained by methodological differences. On the one hand, whereas Palazova et al. (2013) used a standard LDT, Kanske and Kotz (2007) employed a visual hemifield LDT. On the other hand, whereas the experimental stimuli of Kanske and Kotz (2007) were nouns, Palazova et al. (2013) tested verbs and it is well-known that nouns and verbs are differently processed (see for instance, Van Assche et al., 2013). Whatever the reason for the discrepancies, what is relevant is that the above studies failed to support the proposal of Kousta et al. (2011).

Kousta et al. (2011) also posited that the emotional content of words would play a role during their acquisition, facilitating the acquisition of abstract concepts. Our results suggest that this facilitation is also observed when people learn vocabulary in a new language. The modulation of the effects of the emotional content of words by their concreteness in the direction predicted by Kousta et al. (2011) found in this work contrasts with the results of the above discussed studies (Kanske and Kotz, 2007; Palazova et al., 2013). It might be that this modulation is not apparent when vocabulary is firmly established. Rather, it would be more easily observed during (first or foreign) language acquisition. During this process, emotional content might help to acquire those words that are harder to learn, namely abstract words.

Concerning the mechanism involved in the beneficial effects of emotional content, we should take into consideration that acquiring foreign words by means of a paired associate learning involves the assignment of a new name to an already existing concept. The results of this study and of past research suggest that there is something about the representation of some types of L1 words that makes it easier to attach a new name onto it (see De Groot, 2011). This would be the case of concrete words with respect to abstract words. Different proposals have been made with respect to these representational differences. For instance, according to the dual-code theory (Paivio, 1986), semantic representations are richer for concrete words than for abstract words, because only the former are represented in a non-verbal system besides being represented in a verbal system. Alternatively, Schwanenflugel and Shoben (1983) considered that concrete words have stronger and denser associations to contextual knowledge than abstract words. Finally, from a connectionist point of view, Plaut and Shallice (1993) argued that the representation of concrete words is supported by a higher number of units or semantic features than the representation of abstract words. All these proposals assume that the representation of concrete words is richer than that of abstract words and this is the cause of the disadvantage for the latter in processing. The emotional content, whatever the mechanism involved – affectively valenced words having higher semantic richness (e.g., Hofmann et al., 2009), capturing more attentional resources (Pratto and John, 1991), being prioritized in the process of binding to the context (Mackay et al., 2004), or being more elaborately processed during encoding (Sharot and Phelps, 2004), would reduce the disadvantage for abstract words in the process of labeling an existing concept with a novel word.

A final relevant result of the present work refers to valence effects. We obtained a more robust emotional advantage for negative abstract words than for their positive counterparts. Indeed, in the LDT only negative but not positive abstract words did show an advantage in processing. In the translation task, although both types of words were better translated than abstract neutral words, the advantage was higher for negative words than for positive ones. In our opinion, a possible explanation for the superiority of negative words is related to their adaptive function. According to Pratto and John (1991), negative stimuli contain more survive-relevant information than positive stimuli. For that reason, they would be preferentially attended (i.e., the so-called negativity bias) and, as a consequence, better remembered than positive and neutral words. Regarding the novel-word learning paradigm applied in this research, the preferential attention to negative words during the learning phase might have facilitated the formation of links between the Basque words and both their translation equivalents and existing concepts. We would like to note that the grammatical class of the stimuli in the present study might have contributed also to this negativity bias. Most of the experimental words were names. If we had used adjectives instead of nouns, a different pattern of findings might have been observed. Indeed, a positivity advantage is often reported for lists consisting of adjectives (Herbert et al., 2008), a possible reason for this advantage being the high self-relevance of adjectives. Regardless of the cause of the superiority for negative words, it is worth noting that the results obtained in the translation task suggest that this advantage is particularly strong in the first session, in which the percentage of correctly translated negative abstract words almost duplicated that of neutral and positive words. This superiority is observed in both sessions, but its magnitude is reduced over time. In contrast, the advantage of positive abstract words over neutral words, although slower, is more stable across sessions. This result is in line with other studies in the emotional memory literature demonstrating that the superiority for negative words in immediate memory tests decreases after a delay of several hours or days, whereas this decrease is much less pronounced for positive words (e.g., Toyama et al., 2014). Our findings, as those of Toyama et al. (2014), might be accounted for by the so-called fading bias effect, according to which the intensity of the negative affect produced by any event fades faster than that of positive emotion (e.g., Walker and Skowronski, 2009). This phenomenon has been described in the field of autobiographical memory and has only recently been applied to the study of emotional verbal information (Toyama et al., 2014).

To conclude, the present study shows that emotional content facilitates the acquisition of new vocabulary and that this effect is modulated by concreteness as well as by word valence. Our findings suggest that emotional content is a relevant variable to consider in novel-word learning studies. Furthermore, our results can have a practical application, as they demonstrate that foreign abstract neutral words (i.e., those lacking affective charge) are the hardest to learn. Therefore, teaching strategies should be directed to improve the acquisition of those words in foreign language training programs.

## Conflict of Interest Statement

The authors declare that the research was conducted in the absence of any commercial or financial relationships that could be construed as a potential conflict of interest.

## Footnotes

1 We thank Dr. Herbert for this suggestion.
