# Supplementary material for: The role of emotionality in the acquisition of new concrete and abstract words
Source: Front Psychol. 2015 Jul 9;6:976. doi: 10.3389/fpsyg.2015.00976 (PMC4497307; doi:10.3389/fpsyg.2015.00976)
Supplement: Supplementary file 1 [file Data_Sheet_1.DOCX]

Appendix: Basque words used as experimental stimuli and their Spanish and English translations

|  | Basque | Spanish | English |  |
| --- | --- | --- | --- | --- |
| Conditions |  |  |  |  |
| Concrete positive | abesti | canción | song |  |
|  | besarkada | abrazo | hug |  |
|  | txapeldun | campeona | champion |  |
|  | bihotz | corazón | heart |  |
|  | olerki | poesía | poetry |  |
|  | urtebetetze | cumpleaños | birthday |  |
|  | txirrista | tobogán | Slide |  |
|  | hegazkin | avión | airplane |  |
| Concrete negative | hilkutxa | ataúd | coffin |  |
|  | gelaxka | celda | cell |  |
|  | ebasle | ladrón | thief |  |
|  | sastakai | puñal | dagger |  |
|  | minbizi | cáncer | cancer |  |
|  | odol | sangre | blood |  |
|  | amildegi | precipicio | precipice |  |
|  | eroetxe | manicomio | madhouse |  |
| Concrete neutral | bulego | oficina | office |  |
|  | artelazki | corcho | cork |  |
|  | inurri | hormiga | ant |  |
|  | lanpas | felpudo | doormat |  |
|  | igogailu | ascensor | elevator |  |
|  | pospolo | cerilla | match |  |
|  | galtzerdi | calcetín | sock |  |
|  | apalategi | estantería | shelves |  |
| Abstract positive | berotasun | entusiasmo | enthusiasm |  |
|  | arrakasta | éxito | success |  |
|  | itxaropen | esperanza | hope |  |
|  | heziketa | educación | education |  |
|  | askatze | liberación | liberation |  |
|  | gainditze | superación | overcoming |  |
|  | erdiespen | logro | achievement |  |
|  | sustagarri | incentivo | incentive |  |
| Abstract negative | norberekeria | egoísmo | egoism |  |
|  | hondamen | quiebra | bankruptcy |  |
|  | purrustada | desprecio | contempt |  |
|  | erruduntasun | culpabilidad | culpability |  |
|  | ukatze | rechazo | rejection |  |
|  | aiher | rencor | rancor |  |
|  | beldur | temor | fear |  |
|  | larri | grave | severe |  |
| Abstract neutral | kudeaketa | gestión | management |  |
|  | erabilera | manejo | handling |  |
|  | hautagai | candidato | candidate |  |
|  | eskari | instancia | request |  |
|  | igarotze | tránsito | traffic |  |
|  | erantzun | réplica | replica |  |
|  | itxura | apariencia | appearance |  |
|  | erakunde | institución | institution |  |
